# Association Analysis of* NALCN* Polymorphisms rs1338041 and rs61973742 in a Chinese Population with Isolated Cervical Dystonia

**DOI:** 10.1155/2016/9281790

**Published:** 2016-04-28

**Authors:** Qingqing Zhou, Jing Yang, Bei Cao, Yongping Chen, Qianqian Wei, Ruwei Ou, Wei Song, Bi Zhao, Ying Wu, Huifang Shang

**Affiliations:** Department of Neurology, West China Hospital, Sichuan University, Chengdu, Sichuan 610041, China

## Abstract

*Background*. A genome-wide association study (GWAS) demonstrated a possible association between cervical dystonia (CD) and a sodium leak channel, nonselective (*NALCN*) gene. However, the association between *NALCN* and CD was largely unknown in Asian population. The present study was carried out to examine the associations between the two single nucleotide polymorphisms (SNPs) rs1338041 and rs61973742 in the* NALCN* gene and CD in a Chinese population.* Methods*. In a cohort of 201 patients with isolated CD, we genotyped the two SNPs rs1338041 and rs61973742 using polymerase chain reaction restriction fragment length polymorphism (PCR-RFLP). We also included 289 unrelated, age- and sex-matched healthy controls (HCs) from the same region.* Result*. No significant differences were observed in either the genotype distributions or the minor allele frequencies (MAFs) of the two SNPs between the CD patients and the HCs. There were no significant differences between early-onset and late-onset CD patients, between patients with and without a positive family history of dystonia, or between patients with and without tremor or sensory tricks.* Conclusion*. Lack of association between the SNPs of *NALCN* and CD suggests that the SNPs of* NALCN* do not play a role in CD in a Chinese population.

## 1. Introduction

Dystonia is a movement disorder characterized by involuntary sustained or intermittent muscle contractions affecting one or more sites of the body causing abnormal, often repetitive movements, postures, or both [1]. Cervical dystonia (CD), the most common form of late-onset focal dystonia, is characterized by sustained or intermittent neck muscle contractions causing abnormal head movements and postures [2, 3].

Up to now, more than 20 loci (from* DYT1* to* DYT25*) have been identified to be the causative loci of primary dystonia [4] after the first identified* TOR1A* (*DYT1*) gene linked to early-onset primary dystonia [5, 6]. Among them,* CIZ1* (*Cip1-interacting zinc finger protein; DYT23*),* ANO3 *(*anoctamin 3; DYT24*), and* GNAL* (*guanine nucleotide binding protein, alpha activating activity polypeptide, olfactory type; DYT25*) genes have been discovered for CD [7–9]. However, mutations of these genes mainly are seen in familial CD patients [4, 10]. Although most of patients with late-onset dystonia often seem to be sporadic patients, it appears to have a strong genetic basis [11]. Unfortunately, at present, the genetic architecture of late-onset dystonia remains largely unknown [10].

A recent genome-wide association study (GWAS) has been performed on British patients with focal CD. In this study, although no loci reached a statistically significant association with CD, the *P* value of the single nucleotide polymorphism (SNP) rs1338041, which is located in an intron region of sodium leak channel, nonselective (*NALCN*) gene on chromosome 13, was rather low [12]. After subsequent imputation, the results showed a few clusters with potential significance. Rs61973742 within the 5′ untranslated region (UTR) in the* NALCN* was one of the most meaningful SNPs [12]. However, a replicated study of the GWAS from Spain found no association between these two SNPs of* NALCN*—rs1338041 and rs61973742—with CD [13].

As we all know, GWAS is an efficient method to understand genetic underpinnings of genetic complicated disease that are not based on prior knowledge; however, false positive results may be produced in the meantime [14]. Therefore, it is necessary to confirm the finding of GWAS in other ethnic groups. In the current study, we investigated the association of the most potential significance SNPs (rs1338041 and rs61973742) of* NALCN* with CD in a Chinese population.

## 2. Methods

### 2.1. Subjects

A total of 201 patients with focal cervical dystonia were recruited from the department of neurology at West China Hospital of Sichuan University. Patients were examined and diagnosed by movement disorder specialists according to the current criteria [15]. Patients carrying GAG deletion mutation of the* DYT1* gene and having a known causes, including traumatic or structural brain lesions, or treatment with neuroleptic drugs or patients diagnosed with another syndrome with dystonia, such as Parkinson's disease or dystonia plus syndrome, were excluded from the study. Patients with positive family history of dystonia were defined as having one or more first-degree or second-degree family members with dystonia as previously described [16]. A total of 289 unrelated healthy individuals (59.9% women; mean age 43.35 ± 14.77 years) from the same areas of residence were recruited to the study as the healthy controls (HCs) group. None of the HCs had neurological disorders or psychiatric disorders examined by neurologists. Witten informed consent was obtained from all participants before being enrolled, and the study was approved by the Ethics Committee of Sichuan University.

### 2.2. Genotyping

Peripheral blood samples were collected from all participants. Genomic DNA was extracted from peripheral blood leukocytes using standard phenol-chloroform procedures. Genotype for rs1338041 and rs61973742 was performed by polymerase chain reaction restriction fragment length polymorphism (PCR-RFLP) analysis. Two SNPs were all amplified using a forward mismatched primer that creates a restriction enzyme digestion site and the primer sequences and the PCR conditions of two SNPs are shown in Supplementary Table  1, in Supplementary Material available online at http://dx.doi.org/10.1155/2016/9281790. PCR products of the two SNPs were all digested with DraI (New England Biolabs, USA). The RFLP results were confirmed by direct sequencing of the PCR products.

### 2.3. Statistical Analysis

The genotype and minor allele frequencies (MAFs) between patients and controls were analyzed using a standard chi-square test or Fisher's exact test. The comparison of continuous data was assessed by Student's *t*-test. The results of all continuous data are presented as the mean ± standard deviation (SD), and a two-tailed *P* < 0.05 was considered statistically significant. Deviation from Hardy-Weinberg equilibrium (HWE) in controls was tested. Statistical analysis was performed using SPSS version 19.0 (SPSS, Chicago, IL, USA). The power was calculated by the Quanto software (Version 1.2.4; USC, Los Angeles, CA) [13]. The Bonferroni correction for multiple comparisons was performed if necessary.

## 3. Results

The mean age of 201 patients with CD was 41.25 ± 15.76 years at examination time, and the mean age of onset of patients was 38.27 ± 16.06 years. Among all CD patients, the ratio of male to female was 77/124. Fifty patients (24.9%) had an age of onset less than 26 years, and forty-one patients (20.4%) had a positive family history of dystonia.

The genotype frequency distributions in the controls did not deviate significantly from the Hardy-Weinberg equilibrium (0.522 and 0.464 for rs1338041 and rs61973742, resp.). No significant differences were found in the genotype distributions or MAFs of the two SNPs between CD patients and controls (Table 1). No differences were observed in the genotype distributions or MAFs between patients with early-onset and late-onset CD or between patients with and without a family history of dystonia (Table 2). There were no differences in the genotype distributions or MAFs in terms of tremor or sensory tricks (Table 3).

## 4. Discussion

This is the first study on the genetic susceptibility of the rs1338041 and rs61973742 SNPs of* NALCN* and CD in a Chinese population. In the current study, rs1338041 and rs61973742 SNPs were not found to modify the susceptibility to CD.

The* NALCN* gene comprises at least 44 exons (43 coding exons) encoding NALCN, which is a voltage-independent and cation-nonselective channel, and is mainly responsible for the leaky sodium transport across neuronal membranes and controls neuronal excitability [17, 18]. A previous GWAS showed the possible association between the two SNPs of* NALCN* and CD in 212 British resident CD patients of European descent. Given that ion channels are crucial components of cellular exciting ability and are involved in many neurological diseases, for example,* ANO3*, whose mutations have detected in sporadic CD, functions as calcium activated chloride channels which modulate neuronal excitability [19]. It supports that an ion channel may be a potential candidate gene for dystonia. Therefore, the British study came to the following conclusion:* NALCN*, whose encoded protein belongs to a Na^+^-leak channel, may be a plausible candidate gene for dystonia [12].

However, our current study and the Spanish study both failed to replicate the association of rs1338041 and rs61973742 with CD [12, 13]. Several factors should be considered to explain such difference. First of all, in genetic analysis, ethnic population specificity must be taken into consideration as an essential factor. For example, according to the British GWAS, the minor A-allele of rs1338041 showed a doubtful risk to CD patients for the fact that the frequency of minor A-allele seemed higher in CD patients than controls, while the Spanish population and the present study both manifested a higher frequency of A-allele in controls than in patients, meaning a possible protective effect of A-allele. In the case of rs61973742, this phenomenon does not exist, the potential effects of these three available studies making no difference. Secondly, given that the clinical manifestation of dystonia is diversity, the patients recruited among studies could be different. However, our study is in accordance with previous studies on British or Spanish, both containing focal CD only [12, 13]. Thirdly, a major difference is that the age of onset of CD in our patients (38.27 ± 16.06 years) was much less than the other two studies (British: 60.6 ± 10.9 years, and Spanish: 43.5 ± 15.7 years). Considering this factor, we divided our patients into two groups, early onset and late onset. However, no differences in the genotype or allele frequencies between early-onset and late-onset CD were found either. Therefore, these factors may contribute little to the differences.

Finally, the sample size must be taken into consideration. The British GWAS reported a putative association of CD with SNPs in the* NALCN*. However, there was no single SNP that reached the statistical significance in that no single SNP passed the genome-wide significance level (defined as *P* < 5 × 10^−8^) after GWAS. The possible reason is that the sample size is not enough to discover some SNPs with small effect on the disease according to the author. The prevalence of primary dystonia in China was 27.0 per million persons based on the minimum estimates [20]. Therefore, the present study had a probability of 80% power to detect genetic effects at an OR of 1.85 under an additive model in our sample (two-sided, *P* < 0.01) [13]. The Spanish study also had a strong power to find the association between SNPs of* NALCN* and CD [13]. To sum up, it seems that variations in the* NALCN* gene might not be associated with CD. The British GWAS might overvalue the positive effect of SNPs of the* NALCN* gene in dystonia [12].

In conclusion, the lack of association between* NALCN* SNPs rs1338041 and rs61973742 and CD suggested that SNPs of the* NALCN* gene do not play a role in Chinese CD population.

## Supplementary Material

The primer sequences, the length of the PCR products and the restriction enzymes used to analyze the SNPs rs1338041 and rs61973742.

## Figures and Tables

**Table 1:** Distribution of genotype and allele frequency in cervical dystonia patients and controls.

	Genotype				MAF		
rs61973742	AA (%)	AG (%)	GG (%)	*P* value	G (%)	*P* value	OR (95% CI)

CD (*N* = 201)	135 (67.2)	63 (31.3)	3 (1.5)	0.189	69 (17.2)	0.110	1.305 (0.941–1.809)
HC (*N* = 289)	177 (61.2)	101 (35.0)	11 (3.8)	0.102^#^	123 (21.3)	0.068^#^	1.360 (0.978–1.891)^#^

rs1338041	AA (%)	AC (%)	CC (%)	*P* value	A (%)	*P* value	OR (95% CI)

CD (*N* = 201)	36 (17.9)	100 (49.8)	65 (32.3)	0.145	172 (42.8)	0.056	0.779 (0.603–1.007)
HC (*N* = 289)	72 (24.9)	139 (48.1)	78 (27.0)	0.051^#^	283 (49.0)	0.073^#^	0.790 (0.611–1.022)^#^

CD: cervical dystonia; HC: health control; MAF: minor allele frequency; OR: odds ratio; CI: confidence interval.

^#^Adjusted for sex and age.

**Table 2:** Distribution of genotype and allele frequency in different clinical feature of cervical dystonia patients.

Variations	rs61973742					rs1338041				
	AA, AG, GG	*P* value	MAF (%)	*P* value	OR (95% CI)	AA, AC, CC	*P* value	MAF (%)	*P* value	OR (95% CI)
Sex										
Men (*N* = 77)	49, 26, 2	0.476	30 (19.5)	0.332	0.771 (0.456–1.304)	16, 41, 20	0.295	73 (47.4)	0.140	1.356 (0.904–2.035)
Female (*N* = 124)	86, 37, 1		39 (15.7)			20, 59, 45		99 (39.9)		
Family history										
Positive (*N* = 41)	25, 15, 1	0.590	17 (20.7)	0.337	0.742 (0.403–1.367)	6, 18, 17	0.370	30 (36.6)	0.203	0.723 (0.438–1.193)
Negative (*N* = 160)	110, 48, 2		52 (16.3)			30, 82, 48		142 (44.4)		
Age at onset										
<26 (*N* = 50)	37, 11, 2	0.077	15 (15.0)	0.508	1.234 (0.662–2.300)	8, 26, 16	0.902	42 (42.0)	0.854	0.958 (0.660–1.514)
>26 (*N* = 151)	98, 52, 1		54 (17.9)			28, 74, 49		130 (43.0)		

MAF: minor allele frequency; OR: odds ratio; CI: confidence interval.

**Table 3:** Distribution of genotype and allele frequency in cervical dystonia patients with or without special clinical features.

Variations	rs61973742					rs1338041				
	AA, AG, GG	*P* value	MAF (%)	*P* value	OR (95% CI)	AA, AC, CC	*P* value	MAF (%)	*P* value	OR (95% CI)
Sensory trick										
Yes (*N* = 177)	119, 55, 3	0.802	61 (17.2)		0.961 (0.428–2.154)	33, 86, 58	0.627	152 (42.9)	0.867	1.053 (0.527–1.941)
No (*N* = 24)	16, 8, 0		8 (16.7)	0.922		3, 14, 7		20 (41.7)		
Tremor										
Yes (*N* = 108)	71, 36, 1	0.684	38 (17.6)		0.937 (0.556–1.527)	14, 54, 40	0.091	82 (38.0)	0.035^*∗*^	0.653 (0.438–0.972)^*∗*^
No (*N* = 93)	64, 27, 2		31 (16.7)	0.806		22, 46, 25		90 (48.4)		

MAF: minor allele frequency; OR: odds ratio; CI: confidence interval.

^*∗*^No significant differences after the Bonferroni correction.
