# Supplementary material for: Association Analysis of NALCN Polymorphisms rs1338041 and rs61973742 in a Chinese Population with Isolated Cervical Dystonia
Source: Parkinsons Dis. 2016 Apr 28;2016:9281790. doi: 10.1155/2016/9281790 (PMC4864546; doi:10.1155/2016/9281790)
Supplement: Supplementary file 1 — The primer sequences, the length of the PCR products and the restriction enzymes used to analyze the SNPs rs1338041 and rs61973742. [file 9281790.f1.pdf]

Supplementary table: The primer sequences, the length of the PCR products and the restriction enzymes used to be analyze SNPs

SNPs	Primers	PCR products(bp)	Restriction enzyme	Fragments(bp)
rs61973742	F:5' GTAAAACGACGGCCAGTCCCATAAAATGCACTTAAATATT 3'★<br>R: 5' CAAAGATGTCAGTTACTCACAGG 3'	232	DraI	41+191
rs1338041	F: 5' GTAAAACGACGGCCAGTTTTGTAAACACCAGGCTATATGT 3' ★<br>R: 5' TACAGATGAGGAAACTTAGGCA 3'	168	DraI	42+126

★primers were mismatched to make a restriction enzyme site.
